# 3D reconstruction of proximal femoral fracture from biplanar radiographs with fractural representative learning

**DOI:** 10.1038/s41598-023-27607-2

**Published:** 2023-01-09

**Authors:** Danupong Buttongkum, Pairat Tangpornprasert, Chanyaphan Virulsri, Numphung Numkarunarunrote, Chavarin Amarase, Thananop Kobchaisawat, Thanarat Chalidabhongse

**Affiliations:** 1 Center of Excellence for Prosthetic and Orthopedic Implant, Chulalongkorn University, Bangkok, 10330 Thailand; 2 Department of Mechanical Engineering, Faculty of Engineering, Chulalongkorn University, Bangkok, 10330 Thailand; 3 Biomedical Engineering Research Center, Faculty of Engineering, Chulalongkorn University, Bangkok, 10330 Thailand; 4 Perceptual Intelligent Computing Lab, Department of Computer Engineering, Faculty of Engineering, Chulalongkorn University, Bangkok, 10330 Thailand; 5 Applied Digital Technology in Medicine Research Group, Chulalongkorn University, Bangkok, 10330 Thailand; 6 Department of Radiology, Faculty of Medicine, Chulalongkorn University, Bangkok, 10330 Thailand; 7 Hip Fracture Research Unit, Department of Orthopaedics, Faculty of Medicine, Chulalongkorn University, Bangkok, 10330 Thailand

**Keywords:** Biomedical engineering, Computer science, Computational science

## Abstract

A femoral fracture is a severe injury occurring in traumatic and pathologic causes. Diagnosis and Preoperative planning are indispensable procedures relying on preoperative radiographs such as X-ray and CT images. Nevertheless, CT imaging has a higher cost, radiation dose, and longer acquisition time than X-ray imaging. Thus, the fracture 3D reconstruction from X-ray images had been needed and remains a challenging problem, as well as a lack of dataset. This paper proposes a 3D proximal femoral fracture reconstruction from biplanar radiographs to improve the 3D visualization of bone fragments during preoperative planning. A novel Fracture Reconstruction Network (FracReconNet) is proposed to retrieve the femoral bone shape with fracture details, including the 3D Reconstruction Network (3DReconNet), novel Auxiliary class (AC), and Fractural augmentation (FA). The 3D reconstruction network applies a deep learning-based, fully Convolutional Network with Feature Pyramid Network architecture. Specifically, the auxiliary class is proposed, which refers to fracture representation. It encourages network learning to reconstruct the fracture. Since the samples are scarce to acquire, the fractural augmentation is invented to enlarge the fracture training samples and improve reconstruction accuracy. The evaluation of FracReconNet achieved a mIoU of 0.851 and mASSD of 0.906 mm. The proposed FracReconNet’s results show fracture detail similar to the real fracture, while the 3DReconNet cannot offer.

## Introduction

A femoral fracture is a severe injury occurring in any age, gender, nationality and geography. Traumatic causes such as road accidents, falling from a height, and pathological causes such as osteoporosis result in the occurrence of fractures^1^. Fracture of the proximal femur is one of the most frequent fractures because its anatomical shape and load-bearing cause high mechanical stress concentration^2^. According to a worldwide study, the number of fractures is projected to increase from 2.1 million in 2005 to over 3 million fractures in 2025^1,3^. Patients subjected to fracture need to be treated as soon as possible to avoid complications like nonunion, avascular necrosis and premature death^4^. The standard treatment for a femoral fracture is closed reduction with internal fixation. This treatment is to manipulate fragmented bones back to the normal alignment without cutting the skin open. Then internal fixation such as intramedullary nails and dynamic hip screws are inserted to stabilize the fracture, which allows the bones to heal together^4^. During closed reduction, fluoroscopic images (intraoperative radiograph) were taken throughout the procedure to visualize spatial characteristics of fragment bone interpreted by a surgeon. A surgeon who has well experience could accomplish this procedure in 30–45 min while taking a long time for non-experts^5^.

Diagnosis and preoperative planning are crucial procedures for femoral fractures. Preoperative radiographs, such as X-ray images (2D visualization), play an essential role in diagnosis and preoperative planning prior to surgical treatment. Radiologists and surgeons utilize it to visualize fracture characteristics and choose appropriate treatment and fixation devices^4,6^. However, it is tough to interpret spatial characteristics of fractured bones from 2D radiographs that lead the complications after surgery, such as malreduction, nonunion, and fixation failure^7^. 3D imaging for preoperative planning, such as Computed Tomography (CT) and Magnetic Resonance (MR) imaging enormously, provides 3D spatial information on the target anatomical structure^8,9^. A previous investigation using CT images for preoperative planning revealed better clinical outcomes in closed femoral reduction due to early assessed spatial characteristics of bone fragments. The results significantly reduce in the operation time, interoperative radiation frequency, and patient blood loss and show a steeper learning curve for treatment compared to 2D visualization^5,10^. However, CT and MR imaging have long acquisition times, a high budget, high radiation dose (in CT imaging), and high magnetic-field induction (in MR imaging) compared to plain radiograph^11^. Therefore, 3D visualization of patient-specific fragment bone from existing 2D preoperative radiographs would be particularly helpful for enhancing of diagnosis and preoperative planning the treatment.

In recent decades, the Statistical Shape Model (SSM), an atlas-based reconstruction, has competently performed 3D reconstruction tasks for 3D visualization of intact patient-specific anatomy^12,13^. This approach significantly reduced imaging costs and ionizing radiation dose to a patient compared to CT imaging. Because it utilizes only one or two X-ray images to reconstruct a 3D target anatomy^14^. The SSM algorithm utilizes accumulated shape with a statistical spatial characteristic of anatomical structure to be an atlas. The atlas is well-constrained by the geometry of the target anatomy^12^. It is registered to match 2D radiographs using iterative closed point (ICP), minimizing registration errors^13^. Although the SSM is efficient in reconstructing the intact bone, its atlas is incapable of representing various fractural bone shapes due to its mesh surface constraints and time-consuming ICP process. Therefore, it is impractical to apply to the fracture samples directly.

Since 2012, a deep learning-based convolution neural network (CNN) has outperformed image classification tasks and other computer vision applications. A Fully Convolutional Network (FCN) that only performs convolution operations to do pixel-wise classification was the state-of-the-art in semantic segmentation^15^. FCN is trained faster than a fully-connected layer using less number of parameters. They also can handle variable input image size. In addition, a Feature Pyramid Network (FPN) architecture describes a top-down architecture with lateral connections for extracting multi-level feature maps at all scales. As a result, it gains more accuracy and gets rid of gradient vanishing than flattening connection^16,17^. Much research regarding 3D reconstruction from 2D images using these architectures results in high-quality reconstructed 3D volume visually and quantitatively^18,19^. These image-based approaches can directly learn the mapping between 2D images and the 3D volume of patient-specific anatomy without predefined anatomical shapes. Although this method is not so accurate because of the insufficient depth information for a single-view 2D image, the results reveal the characteristics of each sample (such as fractured bone), while the SSM or atlas-based approach does not provide^12,18^. Hence, it is feasible to adopt these techniques to reconstruct fractured bone of patient-specific fractural anatomy with diverse geometry^20^. X2CT-GAN^21^ research employs a Generative Adversarial Network (GAN), which consists of a generator and discriminator fighting against each other to reconstruct the realistic CT image from biplanar chest radiographs. The Generator is FPN constructing the Encoder, Decoder, and Fusion. However, its capability for medical use is unacceptable due to the resulting lack of the lesions. Nowadays, there is no research on the 3D reconstruction of the fractured femoral bone, so it is still unsolved. Therefore, our research will adopt these techniques to encourage 3D visualization of fractural femoral bone preoperatively.

Although the deep learning-based method outperformed in the 3D reconstruction task, this method relies on a large amount of labeled data that is still lacking in the medical imagery domain. Overfitting occurs when there is a lack of sufficient training data. Meanings, a model learns a very high variance function fitting on training data very well but less ability on the test data^22^. The conventional approach deals with this issue by augmenting the existing data. Most augmentation approaches for imagery data employ geometric transformation to existing data such as flipping, cropping, rotation, and translation when task-relevant^22,23^. Although these approaches can increase the diversity of data to avoid an overfitting problem, they can be less capable when dealing with the diversity of fractural bones^4,20,24^. Thus, particular augmentations related to the enlargement of fractural bone samples are needed for training the deep learning framework.

The purpose of this work is to accurately reconstruct the 3D proximal femoral fracture from biplanar radiographs for preoperative planning. Thus, surgeons can utilize 3D visualization of bone fragments to restore anatomical alignment. A novel Fracture Reconstruction Network (FracReconNet) is proposed to retrieve the femoral bone shape with fracture detail including:

I. The 3D Reconstruction Network (3DReconNet), which deploys FCN with FPN architecture used to reconstruct the femoral bone. It can be utilized to encode, decode and fuse biplanar multi-scale features. First, the encoders, which are down-sampling operations with dense connections, extract features and expand of 2D-3D dimensions of each imaging view separately. Second, the decoders, which are up-sampling operations with a lateral encoder concatenation, reveal 3D features from encoded features. Then, the fusion combines decoded features of each imaging view.
II. The novel auxiliary class (AC) is invented to train with bone shapes simultaneously by guiding the network to capture fracture details on the FracReconNet. This class is defined as the fracture representative voxel among each bone fragment.
III. The fractural augmentation approach (FA) is developed to enlarge the diversity of proximal femoral fractures to tackle the shortage of training samples. The proposed network is trained end-to-end with early described techniques.

## Methods

This section presents the fracture reconstruction network (FracReconNet) comprises three modules: the 3D reconstruction network, the auxiliary class, and the fractural augmentation.

### 3D reconstruction network (3DReconNet)

**Figure 1:**
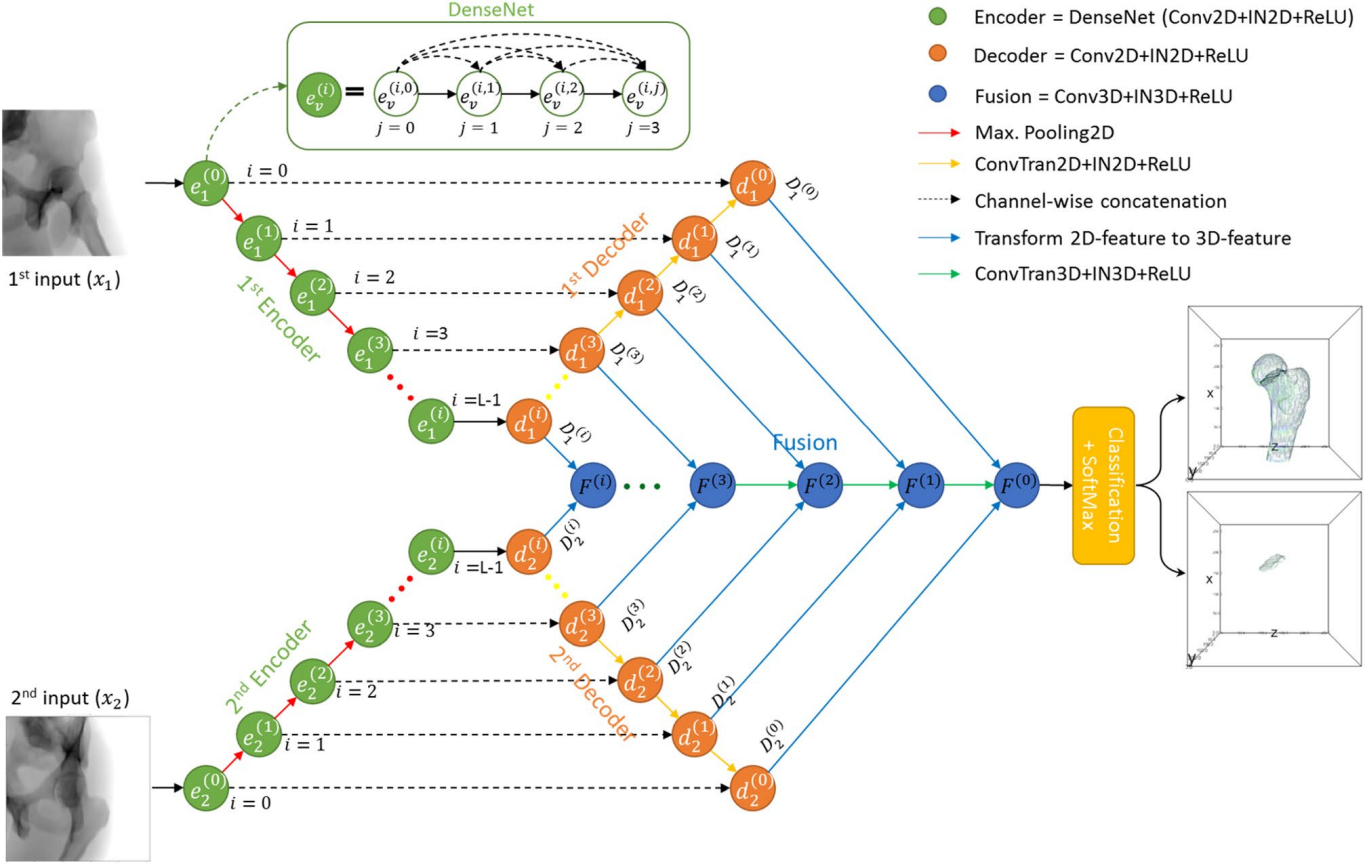
The proposed network for 3D reconstruction of femoral fracture with fracture detail is called 3DReconNet. The network includes Encoder, Decoder, and Fusion modules represented by green, orange, and blue circles. The solid black, red, yellow, and blue arrows indicate 2D convolutional operations, downsampling, upsampling and fusion operations, respectively. While the black dash arrows indicate channel-wise concatenations between encoder and decoder.

The reconstruction network is built based on the fully convolutional network (FCN)^15^ and mimics a feature pyramid network (FPN) architecture^16^. The network includes three modules: Encoder, Decoder, and Fusion (inspired by X2CT-GAN^21^), as shown in Fig. 1. The ground truth is a voxel-based 3D femur shape denoted by $Y$. The input radiographs are denoted as ${x}_{v}$, where the subscript $v \epsilon \left\{\mathrm{1,2}\right\}$ is the view of the input radiograph. Both input views are executed by parallel encoder-decoder modules to generate 3D features. Finally, the biplanar 3D features are combined by the fusion module to obtain desirable 3D femur shapes with fracture details. The details of each module are described in the following sections:

### Encoder module

The encoder learns the features from biplanar images and transfers them to the decoder. First, the encoders extract the features from input images ${x}_{v}$ by a 2D-convolutional operation. To avoid gradient vanishing of a very deep network, the features from the previous operation are passed through DenseNets^25^. It comprises four layers ($0\le j\le 3$), of which each layer includes instance normalization (IN), rectified linear unit (ReLU) activation function, and 2D-convolutional operations (kernel size 3 with same padding and single stride), as shown in Fig. 1 (light green box). Then, the encoded features are down-sampling by a maximum pooling operation (kernel size 2), yielding multi-scale features with 7 abstract levels ($L=7$). Processing all operations yields encoded features ${e}_{v}^{(i)}$, where $0\le i<L$ is the $ith$ abstract level.

### Decoder module

The decoder receives the encoded features ${e}_{v}^{(i)}$ and performs a 2D-convolutional operation (kernel size 3 with same padding and single stride) followed by IN and ReLU activation function, yielding the decoded features ${d}_{v}^{(i)}$. The number of decoded feature channels is prescribed to be the same-scale features at each abstract level to form cubical 3D features. Next, the decoded features are up-sampled by 2D transpose convolutional operation (kernel size 2 and stride 2), then concatenated with the same-scale encoded features. Then the channel dimension of ${d}_{v}^{(i)}$ is transformed into the depth dimension earning 3D decoded features ${D}_{v}^{(i)}$. The 3D features of each view of radiographs will be combined in the fusion module described in the next section.

### Fusion module

**Figure 2:**
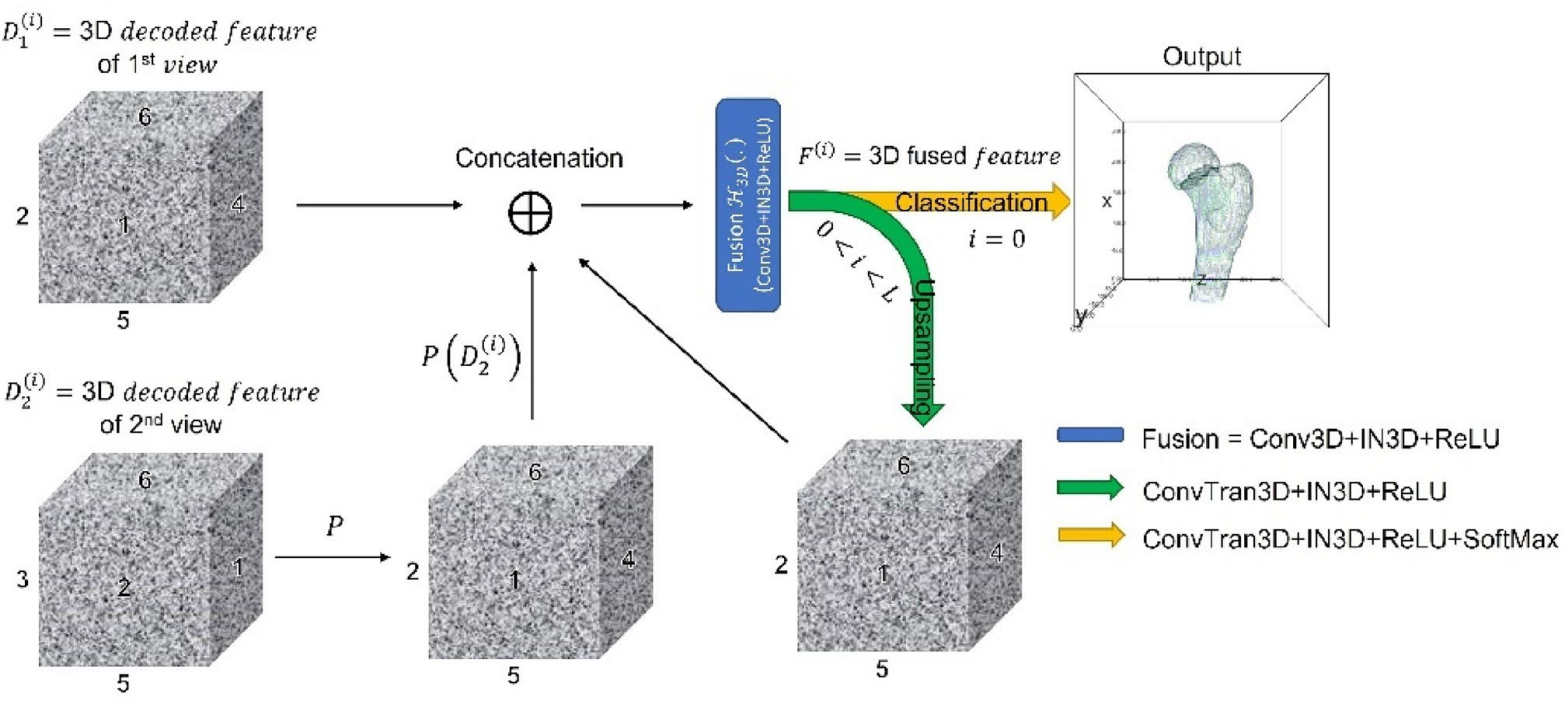
Feature fusion process of biplanar decoded features using convolution over the concatenation of permutation. This diagram explains the fusion process of blue circles in Fig. 1; The symbol $\oplus$ indicates channel-wise concatenation, and the $P$ indicates permutation of the volumetric features.

After the decoding process, we earned a multi-scale of 3D decoded features ${D}_{v}^{(i)}$ of biplanar input images. The 3D decoded features ${D}_{2}^{(i)}$ of the second view are orthogonal with the first view about the human’s vertical axis. So, it needed to be permuted about the vertical axis to conform to the other. After permutation, the 3D decoded features from both views are concatenated. Then the features are performed by a set of 3D convolutional operations (kernel size 3 with same padding and single stride), IN, and ReLU activation function, which yielded 3D fused features ${F}^{(i)}$. Then, the ${F}^{(i)}$ is upsampled by 3D transpose convolution (kernel size 2 and stride size 2) followed by IN and ReLU activation function. The up-sampled features are concatenated with same-scale decoded features for every level (see Eq. 1). The feature fusion process is shown in Fig. 2. The voxel-wise classification is performed at the final layer of fusion by 3D convolutional operation with a kernel size of 1, generating two or three categories of background, bone, and auxiliary class (described in the next section). Finally, the SoftMax function was applied to obtain the probability distribution of each output class.

1 $${F}^{(i)}=\left\{\begin{array}{c}{\Theta }_{3D}\left(\left[{D}_{1}^{\left(i\right)}, P\left({D}_{2}^{\left(i\right)}\right),{\mathcal{U}}_{3D}\left({F}^{\left(i+1\right)}\right)\right]\right), i<L-1\\ {\Theta }_{3D}\left({D}_{1}^{\left(i\right)},P\left({D}_{2}^{\left(i\right)}\right)\right), i=L-1\end{array}\right.$$

where ${\Theta }_{3D}\left(.\right)$ is 3D-convolutional operation followed by IN and ReLU activation function, $P$ is permutation operation, [ ] denotes the channel-wise concatenation and ${\mathcal{U}}_{3D}\left(.\right)$ is 3D-transpose convolutional operation with kernel size 2 × 2 × 2 and stride 2.

### Auxiliary class

Since there is not much research on 3D reconstruction of femoral fracture, the technique dealing with the fracture representation is not available now. Similar research applies conventional CNN-based approaches. Although it has a good reconstruction result, it did not focus on lesions such as fractures^18,21^. Therefore, small fracture detail might be faded out from the result^18^. Our experiment also found the fusion between the close region of each fragment because of highly imbalance between the fracture region and the other regions. This causes misinterpretation of fracture characteristics of 3DReconNet’s result. Nevertheless, from the concept of self-supervised learning, the auxiliary task plays an important role in aiding the network in learning internal representations itself from the synthesis of the existing dataset before the network learns to solve the main task^26–28^. Following the motivation of the auxiliary task, the novel auxiliary class is proposed, which represents the fractural characteristics or mask of the fracture region. It aims to encourage the proposed method of learning internal representation to recognize and reconstruct the fracture.

**Figure 3:**
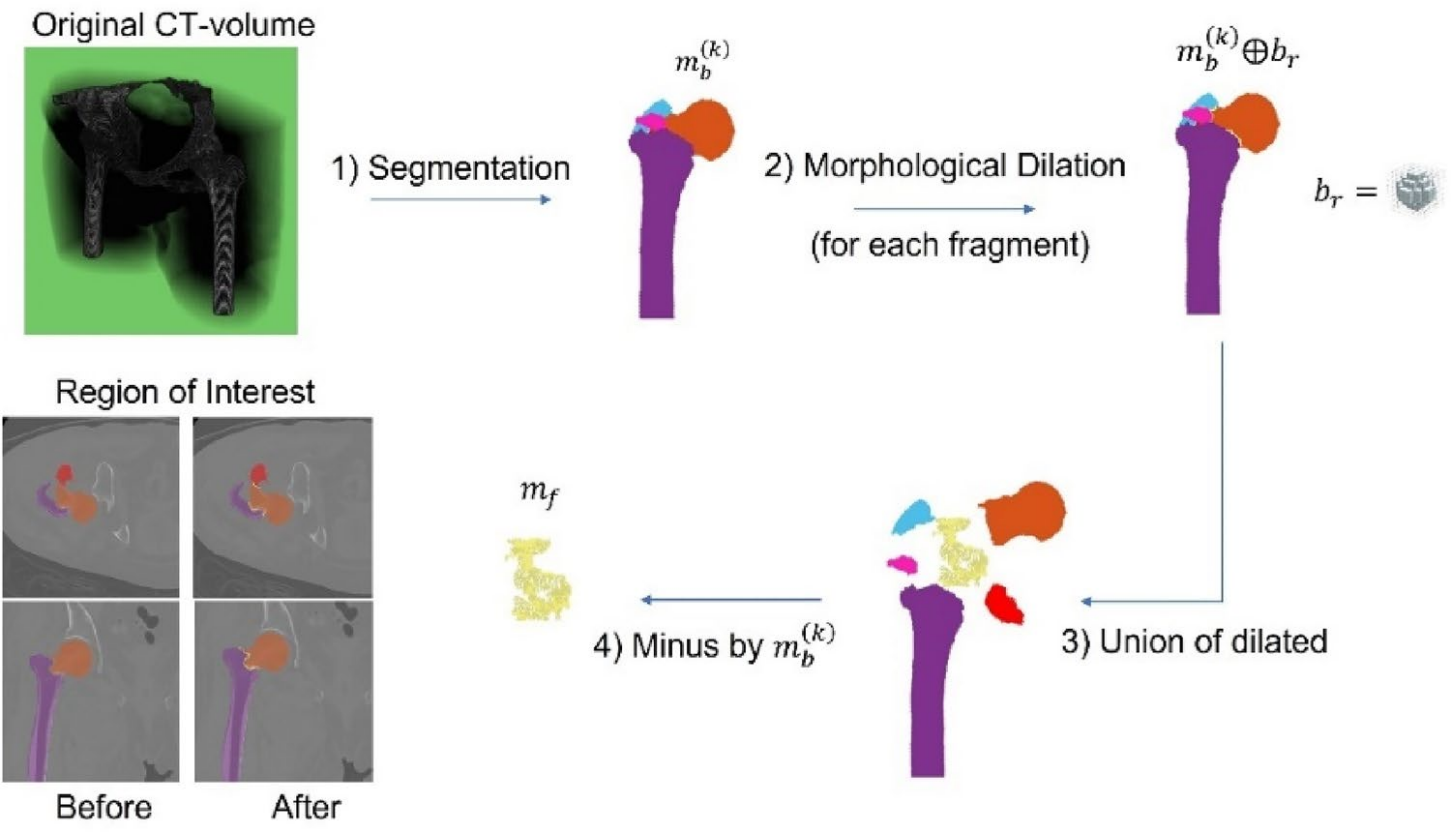
Workflow for synthesis of the Auxiliary class. The workflow includes (1) segmentation of each fragment femur as volume of interest; (2) morphological dilation of each fragment; (3) synthesis auxiliary volume; and (4) define it as the third class of output.

Intensively, the auxiliary class is defined as the fracture representative voxel being among the closest region of each bone fragment and its role to separate any two or more closest fragments of bone away from the others. The auxiliary class is synthesized by morphological technique, as shown in Fig. 3. After the segmentation process (detailed in the dataset section), each fragment of bone (${m}_{b}^{\left(k\right)}$) is annotated to be a different label as $k\in \left\{\mathrm{1,2},\dots ,n\right\}$. Then each fragment is morphologically dilated with a spherical structuring element (${b}_{r}$) with a radius $r=2$ to enlarge the boundary. The intersection of each dilated boundary is united then minus by original each bone fragment, obtaining the auxiliary class (${m}_{f}$) as shown in Eq. (2).

2 $${m}_{f}=\bigcup_{k=1}^{n}\bigcup_{\begin{array}{c}l=1\\ l\ne k\end{array}}^{n}\left({m}_{b}^{\left(k\right)} \oplus {b}_{r}\right)\cap \left({m}_{b}^{\left(l\right)}\oplus{b}_{r}\right)-\bigcup_{k=1}^{n}{m}_{b}^{\left(k\right)}$$

where superscript $k, l\in \left\{1, 2, \dots , n\right\}$ are the $k\mathrm{th}, l\mathrm{th}$ fragment bone, and $n$ is the number of fragment bone. Finally, there are three classes of the training samples, including background, bone, and auxiliary class. Therefore, we can adequately weigh the loss function contributed to background, bone, and auxiliary class to get the best reconstruction result.

### Fractural augmentation

**Figure 4:**
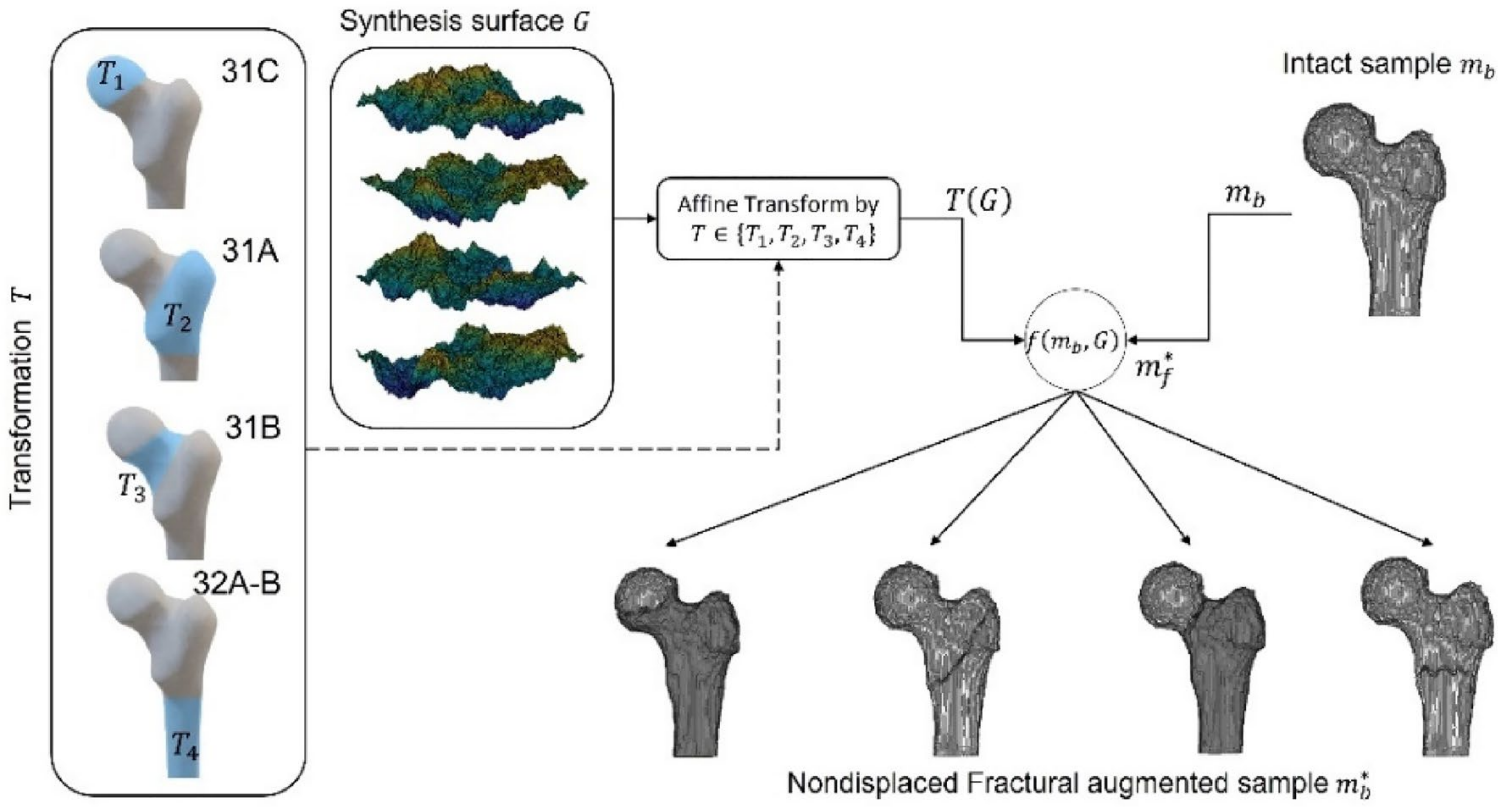
Workflow of the Fractural augmentation. The position of the synthesis surface applied affine transformation is shown in the blue area of the left box. The synthesis surface is randomized every time sampling. The equation $f\left({m}_{b},G\right)$ is described in Eqs. (3)and (4).

For our research, lacking proximal femoral fracture samples as an interesting subject is inevitable. To overcome this limitation, the fractural augmentation approach is presented to enlarge fractural training datasets, as shown in Fig. 4. First, the radially averaged surface from roughness power spectrum (PSD) invented by Kanafi MM. method^29^ is utilized to randomly generate artificial fracture surfaces $G$ (voxel-based mask). Then, for each intact femur sample ${m}_{b}$, the artificial surface mask $G$ is randomly manipulated to the fracture location by affine transformation matric ($T$). The fracture locations are trochanteric (31A), femoral neck nondisplaced (31B), femoral head (31C), and diaphyseal segment fracture (32A–B), according to the AO Foundation and the Orthopaedic Trauma Association^24^ (see Fig. 4). The intersection between generated surface and intact femur is defined as an augmented fracture mask (${m}_{f}^{*}$), as shown in Eq. (3). Furthermore, the augmented bone mask (${m}_{b}^{*}$) is the intact bone mask subtracted by the augmented fracture mask (${m}_{f}^{*}$), as shown in Eq. (4), where the superscript $*$ refers to the augmented sample.

3 $${m}_{f}^{*}={m}_{b}\cap T\left(G\right)$$

4 $${m}_{b}^{*}={m}_{b}-T\left(G\right)$$

Finally, the input radiograph for training the proposed network also needs to reveal fracture detail corresponding to the augmented sample ${m}_{b}^{*}$ and ${m}_{f}^{*}$. To do so, the original intensity volume of the femur ($Q$) needs modification of the intensity to imitate a real fracture sample before taking it to simulate the input radiograph. Lynch et al.^30^ studied the changes in bone intensity at fracture sites, the CT intensity values have dropped from bone intensity by 128 ± 65 in the Hounsfield unit (HU) in the range of soft tissue intensity. Therefore, the original intensity volume $Q$ at the corresponding augmented fracture region $\left(i,j,k\right)\in {m}_{f}^{*}$ must be replaced by randomly sampling soft tissue intensity in the Hounsfield unit, yielding the augmented intensity volume (${Q}^{*}$) (see Eq. 5),

5 $${Q}_{\left(i,j,k\right)}^{*}= \left\{\begin{array}{c}rand\left(\mathrm{63,193}\right), (i,j,k) \epsilon {m}_{f}^{*}\\ {Q}_{\left(i,j,k\right)}, otherwise\end{array}\right.$$

where subscript $\left(i,j,k\right)$ is corresponds to the location in the spatial domain of $Q$ or ${Q}^{*}$. The limitation of this augmentation approach is that it can only enlarge the nondisplaced samples but not the displaced samples.

### Loss function

Since our ground truth mostly includes background class, there is a class imbalance between background, bone and auxiliary classes at an approximate ratio of 9800:200:1, respectively. Therefore, the voxel-wise focal loss is deployed, a variation of cross-entropy loss to tackle this class imbalance problem. The adjustable focusing parameter ($\gamma$) is added into the first term of cross-entropy loss, as illustrated in Eq. (6). It is designed for downing weight contribution of inliers (easy examples) such that they slightly contribute to total loss and mainly focus on outliers (hard example)^31^. The focal loss per class is defined as (Eq. 7)below,

6 $${fl}_{i}(p,y)=\left\{\begin{array}{c} {\left(1-p\right)}^{\gamma }log\left(p\right), y=1\\ \\ {p}^{\gamma }log\left(1-p\right), y=0\end{array} ;i=\left\{0, 1, 2\right\}\right.$$

7 $$FL=\sum_{i = 0}^{2}{w}_{i}{fl}_{i}$$

where ${y}_{i}\in \left\{\mathrm{0,1}\right\}$ specifies the $i\mathrm{th}$ class of ground truth, $p\in \left[\mathrm{0,1}\right]$ is the probability map for class label ${y}_{i}=1$, $\gamma \in \left[\mathrm{0,5}\right]$ is the focusing parameter that smoothly adjusts the rate at which easy examples are down-weighted^31^. The weight ${w}_{i}$ of $i$ class is added before the summation of a total loss to handle the class imbalance. In our experiment, we set $\gamma =2$ and $w=\left[0.15, 0.25, 0.6\right]$ to obtain the best reconstruction result.

## Experiments

### Dataset

Studied retrospectively, CT images were collected from King Chulalongkorn Memorial Hospital, Bangkok, Thailand, which consisted of 132 samples following IRB NO 249/64 (COA No. 541/2021). All participants provided written informed consent before enrollment. The dataset includes 56 intact, 36 nondisplaced, and 40 displaced fracture femur that were demonstrated in Table 1. The CT images cover the pelvic to mid-femoral shaft area. We randomly divide each sample type into training (80%) and test set (20%). The CT images were segmented to obtain the boundary of each femur fragment. Each segmented fragment of the fractural femur is indicated as a voxel-based mask ${m}_{b}^{\left(i\right)}$ (where $i$ indicates fragment number).

**Table 1:** Baseline demographic characteristic of femoral fracture from King Chulalongkorn Memorial Hospital.

Sample type	Male (n = 53) (mean ± SD of age)	Female (n = 79) (mean ± S.D. of age)	Total (n = 132) (mean ± S.D. of age)
Intact	21 (52.37 ± 22.05)	35 (69.23 ± 18.88)	56 (62.91 ± 19.93)
Nondisplaced	14 (58.50 ± 25.79)	22 (64.18 ± 18.80)	36 (61.97 ± 21.43)
Displaced	18 (52.27 ± 20.86)	22 (75.39 ± 15.78)	40 (64.99 ± 17.99)
Overall	53 (53.96 ± 22.43)	79 (69.54 ± 18.31)	132 (63.28 ± 19.68)

The limitation of learning the 3D reconstruction model is lacking pair of corresponding X-ray images and a 3D femur shape. It can be considered expensive and unethical to collect paired data with additional radiation doses to the subject. Thus, we overcome this limitation by simulating a virtual X-ray image named digitally reconstructed radiograph (DRR). The DRR is typically used in medical image processing, such as multimodal registration, atlas-based 3D reconstruction, etc^12,14^. The simulated DRR is similar to the X-ray image compared by a variety of similarity measures such as mutual information, entropy, dice coefficient of intensity histograms overlapping, etc.^32,33^. In this work, to avoid overlapping of contralateral leg appearing in lateral view, the DRRs were simulated in −45° and + 45° of humans’ vertical-axis called the Judet view^34^.

### Experiment setting

The proposed network was trained end-to-end without a pre-trained network. We implemented our network in PyTorch framework^35^. The Adam optimizer was used to optimize our network with learning rate of 1 × 10^–4^ and momentum parameters of ${\beta }_{1}=0.9$ and ${\beta }_{2}=0.99$^36^. Early stopping during the training scheme is also used to avoid overfitting problem, which stops the training when validation loss starts degrading. Our experiments perform on three networks including:

I. 3DReconNet is the 3D reconstruction network (see Fig. 1)
II. 3DReconNet-AC is the 3DReconNet simultaneously trained with the auxiliary class (AC)
III. FracReconNet which is the proposed method is the 3DReconNet-AC network trained with fractural augmented data.

Furthermore, five-fold stratified cross-validation was applied to validate and compare the efficiency of each network. Each type of the sample in the dataset was randomly split into five folds, which were allocated to training set (4 folds) and testing set (onefold).

### Evaluation metrics

We evaluate the performance of our proposed methods using Intersection-over-Union (IoU) and Average symmetric surface distance (ASSD)^37^. The IoU metric is an overlap-based evaluation metric considering volume overlapping between the reconstructed result and ground truth. In contrast, the ASSD is a distance-based evaluation metric considering the average Euclidean distance from a point on the boundary surface of output to ground truth and vice versa. This metric indicates how the boundary surface of the result has achieved boundary surface similarity to ground truth. A Lower ASSD value indicates higher accuracy of the result boundary surface.

### Ethics declarations

Ethics approved by King Chulalongkorn Memorial Hospital, Bangkok, Thailand, following IRB NO 249/64 (COA No. 541/2021). We confirm that all methods were performed in accordance with international guidelines for human research protection as the Declaration of Helsinki, the Belmont Report, CIOMS Guideline, and the International Conference on Harmonization in Good Clinical Practice (ICH-GCP).

## Results

**Figure 5:**
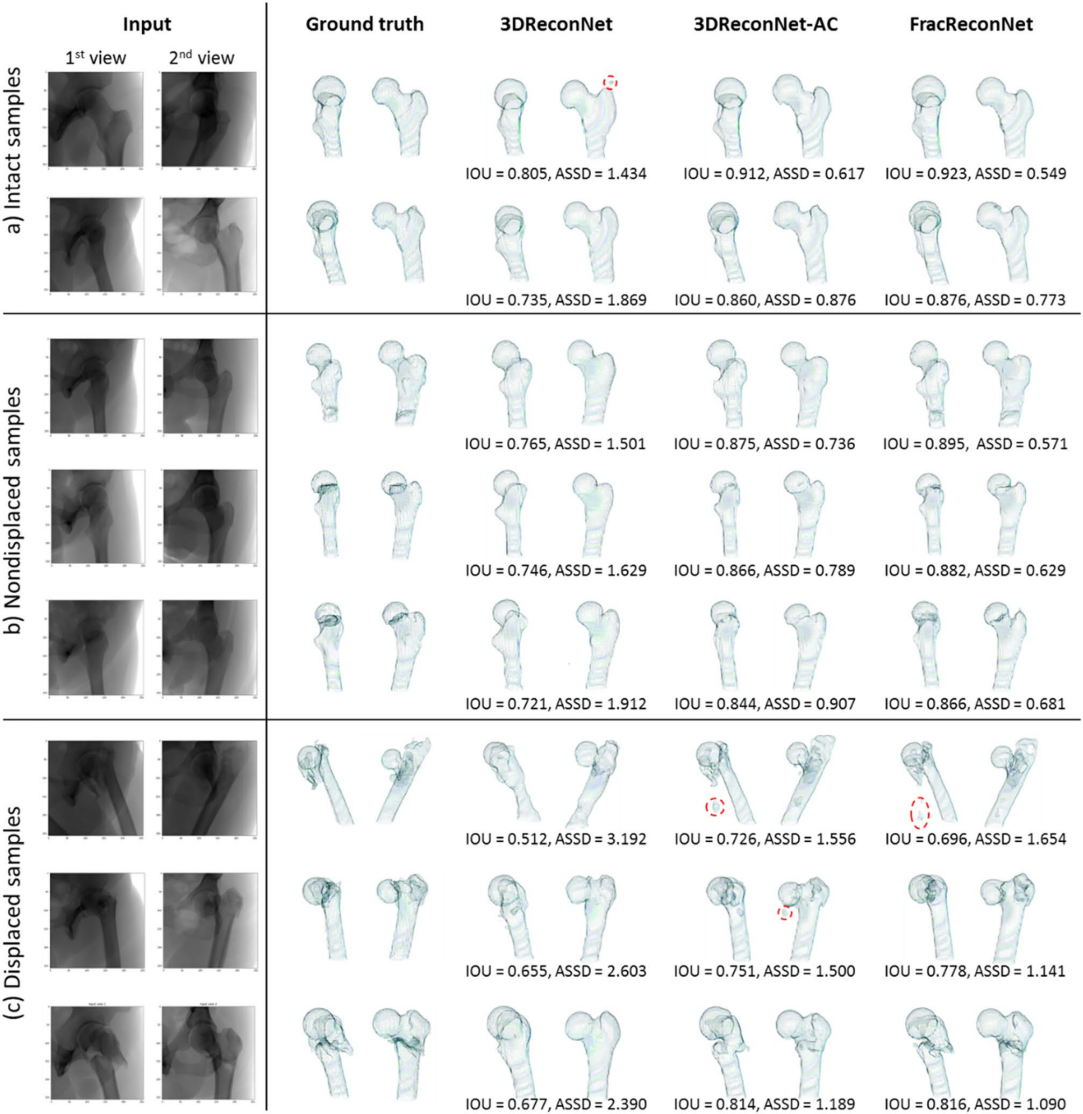
Result of 3D femur reconstruction (**a**) intact (**b**) nondisplaced fracture (**c**) displaced fracture samples. The results are shown in transparent mech surface at −45° and + 45° about the human vertical axis. The red dash circles in the figure indicate bulky noise occurring in the reconstructed results.

**Figure 6:**
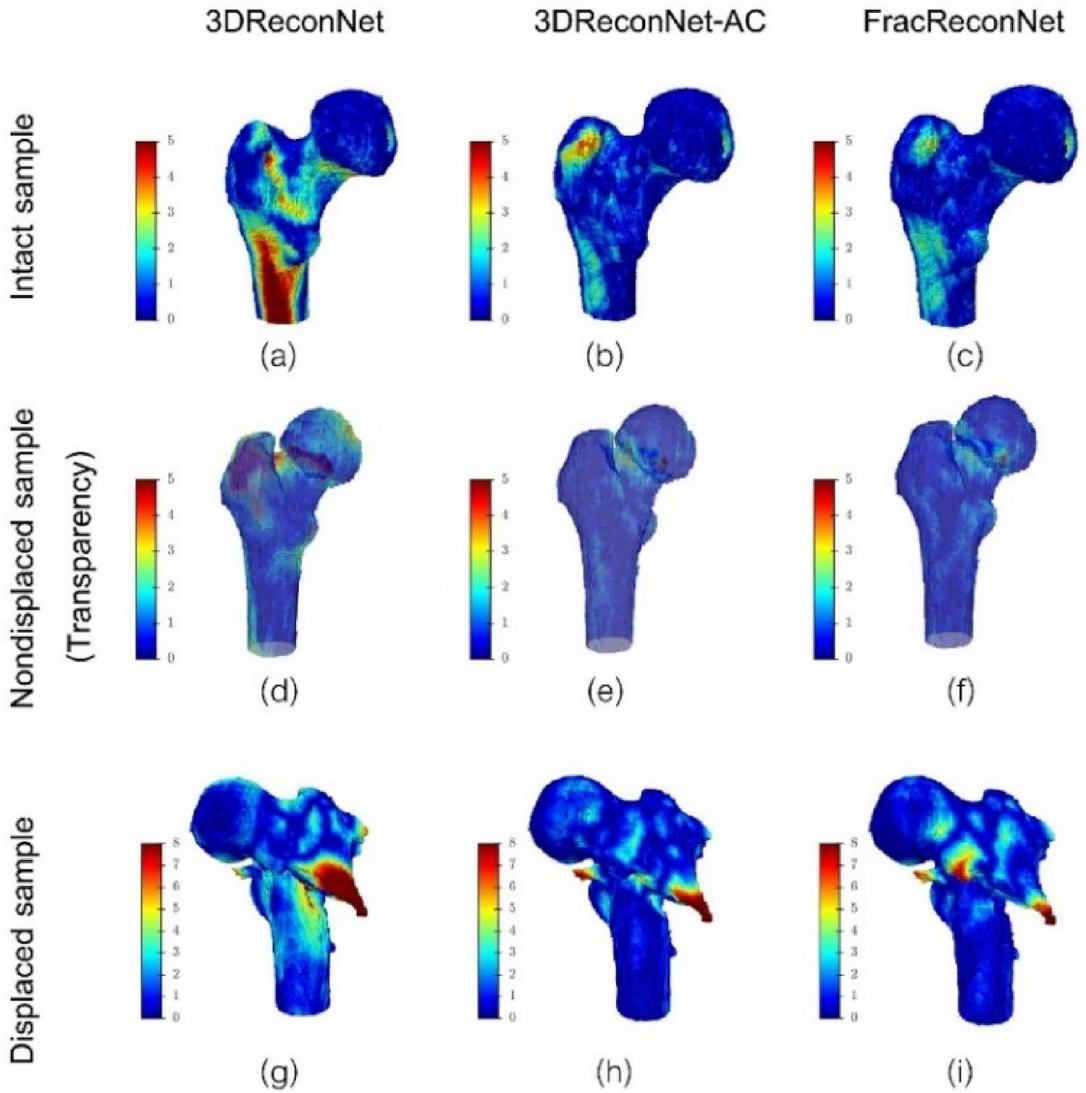
Surface distance error of the reconstructed 3D femur shape using 3DReconNet, 3DReconNet-AC and FracReconNet. The surface distance is based on the ground truth shape for (**a–c**) intact, (**d–f**) nondisplaced, and (**g–i**) displaced samples.

Figure 5 shows transparent wireframe surfaces of the 3D reconstructed result of each network, including the 3DReconNet, 3DReconNet-AC, and FracReconNet in terms of the first and second view corresponding to the input. Figure 6 also shows the surface distance error of the proposed FracReconNet on various sets of sample types, including intact, nondisplaced, displaced fracture, and overall samples. Table 2 also illustrates the quantitative results of five testing folds in terms of the reconstruction accuracy measured by the mean of IoU (mIoU) and ASSD (mASSD) metrics (Value ± SD). For overall samples, the mIoU values were 0.731, 0.841, and 0.851, and the mASSD values were 1.845, 1.070, and 0.906 mm for the 3DReconNet, 3DReconNet-AC and FracReconNet, respectively.

**Table 2:** The quantitative results of the study on the proposed method of various networks.

Method	Evaluation metrics	Sample type			Overall
		Intact	Nondisplaced	Displaced	
3DReconNet	mIoU	0.771 ± 0.080	0.735 ± 0.078	0.657 ± 0.111	0.731 ± 0.101
3DReconNet-AC		0.875 ± 0.036	0.850 ± 0.047	0.772 ± 0.080	0.841 ± 0.069
FracReconNet		0.888 ± 0.034	0.861 ± 0.040	0.779 ± 0.080	0.851 ± 0.070
3DReconNet	mASSD (mm. Unit)	1.556 ± 0.817	1.861 ± 0.651	2.333 ± 0.764	1.845 ± 0.834
3DReconNet-AC		0.889 ± 0.518	1.102 ± 0.639	1.359 ± 0.641	1.070 ± 0.614
FracReconNet		0.754 ± 0.401	0.883 ± 0.363	1.186 ± 0.414	0.906 ± 0.435

It includes 3DReconNet, 3DReconNet-AC and FracReconNet. Mean of the mIoU and mASSD values (mean ± SD) are used as the comparison metrics.

For intact samples (Fig. 5a), the FracReconNet and 3DReconNet-AC assisted by auxiliary class with/without fractural augmentation approach could reconstruct intact bone more accurately compared to 3DReconNet. The complex shape at greater-trochanter and lesser-trochanter were clearly seen. The quantitative results of mIoU and mASSD were (0.771, 1.556 mm) of 3DReconNet, (0.875, 0.889 mm) of 3DReconNet-AC and (0.888, 0.754 mm) of FracReconNet. As seen in Fig. 6c, for the 3DReconNet-AC and FracReconNet, most of the boundary surface error was lower than 1 mm, which was a promising result.

For nondisplaced fracture samples (Fig. 5b), the 3DReconNet could not retrieve fracture details at all. While the 3DReconNet-AC could reconstruct the femur shape with a little fracture detail in some samples. In contrast, the FracReconNet provided better fracture detail. The quantitative results of mIoU and mASSD were (0.735, 1.861 mm) of 3DReconNet, (0.850, 1.102 mm) of 3DReconNet-AC and (0.861, 0.883 mm) of FracReconNet. Although most of the boundary surface error of the nondisplaced sample was lower than 1 mm, the surface distance error at the fracture area was 1–2 mm (see Fig. 6f).

For displaced fracture samples (Fig. 5c), there is a very challenge to reconstruct the fracture that is dislocated from the intact position of each fragment. The 3DReconNet’s results were very distorted compared to its intact shape. Each bone fragment was fused with the others. In contrast, the 3DReconNet-AC and FracReconNet yielded an empirical improvement of the reconstructed shape and the fracture detail. The results of displaced fracture samples showed the separation of each bone fragment clearly. The quantitative results of mIoU and mASSD are (0.657, 2.333 mm) of 3DReconNet, (0.772, 1.359 mm) of 3DReconNet-AC and (0.779, 1.186 mm) of FracReconNet. However, the results still encounter some distortion and some bulky noise, as shown in the red circle in Fig. 5c. There was no difference between the results of the 3DReconNet-AC and FracReconNet. For the surface distance error of FracReconNet (Fig. 6i), the surface error at the intact area was still below 1 mm. But the surface error at the fracture area was worst about 3–5 mm, especially at the tip.

## Discussion

### Performance assessment

The FracReconNet performed well in the reconstruction task. The fusion module (Fig. 2) inside the FracReconNet was capable of combining biplanar decoded features. Performing concatenation of permutation before the convolutional operation, the module learned to fuse each biplanar information by itself, yielding a promising reconstructed shape shown in Fig. 5. The proposed auxiliary class deployed in 3DReconNet-AC and FracReconNet provided more fracture information beyond femur shape so that the network could directly learn the femur shape and its fracture characteristics simultaneously. It gained better mIoU and mASSD values than 3DReconNet. Finally, the fractural augmentation deployed in FracReconNet enlarged the fracture dataset used for training the network. Thus, the network can capture various fracture characteristics resulting in precise fracture detail, especially for nondisplaced samples as shown in Fig. 5b. The artificial surface simulated by Kanafi MM. method^29^ had been proved similar to the fracture region of real fracture samples. The empirical evidence of similarity had been shown via the improvement of the result, especially in nondisplaced fracture samples.

Table 3 presents paired T-test study on five testing folds to test whether each method significantly improved in the reconstruction performance compared to the others. For overlap-based evaluation, the 3DReconNet-AC and the FracReconNet methods have significantly improved the mIoU values about 14.93% and 16.43%, respectively, compared to the 3DReconNet. This improvement was contributed by the novel auxiliary class of fracture characteristics training. The mIoU value of the FracReconNet and the 3DReconNet-AC method was nearly the same. For distance-based evaluation, both 3DReconNet-AC and FracReconNet methods significantly yielded better mASSD than the 3DReconNet method about 41.99% and 50.91%, respectively. The FracReconNet method yielded significantly lower mASSD values than the 3DReconNet-AC method about 15.38%. This improvement illustrated that using the fractural augmentation encourages reducing the mASSD values. The ASSD metric indicated a better improvement in accuracy over the IoU metric (15.38% of mASSD vs 1.30% of mIoU). Since the IoU metric related overlap-based evaluation method had failed to measure the fracture samples, it only accounted for the number of correctly classified voxels of bone class between the result and ground truth without accounting for the fracture voxel (auxiliary class or fracture voxel was merged into the background). Nevertheless, the distance-based ASSD metric takes every point on the boundary, including the fracture surface of the result, into account from the other boundary. Thus, the ASSD metric was more suitable for representing the accuracy of the fracture samples than the IoU metric.

**Table 3:** T-score (P-value) using paired T-Test method comparing various methods.

Method	Baseline			
	3DReconNet	3DReconNet-AC	FracReconNet	Evaluation metrics
3DReconNet		−14.218 (< 0.05)*	−14.065 (< 0.05)*	IoU
3DReconNet-AC	14.218 (< 0.05)*		−2.359 (< 0.05)*	
FracReconNet	14.065 (< 0.05)*	2.359 (< 0.05)*		
3DReconNet		8.260 (< 0.05)*	12.171 (< 0.05)*	ASSD
3DReconNet-AC	− 8.260 (< 0.05)*		2.405 (< 0.05)*	
FracReconNet	− 12.171 (< 0.05)*	−2.405 (< 0.05)*		

The methods include 3DReconNet, 3DReconNet-AC, and FracReconNet, which are compared based on mIoU and mASSD evaluation metrics. Note that * represents a significant difference measured by p-value.

*Significate difference at 95% confident interval of p-value < 0.05.

In preoperative planning usage, deploying the FracReconNet should accommodate surgeons planning the fracture treatment for intact and nondisplaced fracture patients. The results were good enough for anatomical and morphological study due to the surface distance error being lower than 1.0 mm. However, the reconstructed results of the displaced fracture had moderate accuracy (3–5 mm). Therefore, a surgeon who utilizes it for preoperative planning may make the wrong decision. Moreover, the bulky noise occurred in the result, as shown in Fig. 5 (red dash circles). Even if it diminished the quantitative performance (IoU and ASSD value), it does not appear to affect interpretation of preoperative planning.

Moreover, the concept of the auxiliary class and the fractural augmentation could be applied to other fracture locations e.g., Tibia, Humerus, Pelvic e.g., The auxiliary class approach can also be used for other medical purposes, such as for example, liver tumor segmentation, by assigning the tumor as the auxiliary class inside the liver region and then training the model to segment the liver and its tumor. It uses just a single network rather than two cascade networks, as Lei Chen et al. method^38^.

Finally, running time based on NVIDIA GeForce RTX 3090 and RTX 2080Ti graphics cards, the FracReconNet took much more training time than the other method, which took 46 h in total due to further calculation of augmented data. While both 3DReconNet and 3DReconNet-AC took lower training time than the FracReconNet at 14 h. In the testing scheme, every method took 2 h in total. For inference, every method took approximately 2 s per sample.

### Limitations

**Figure 7:**
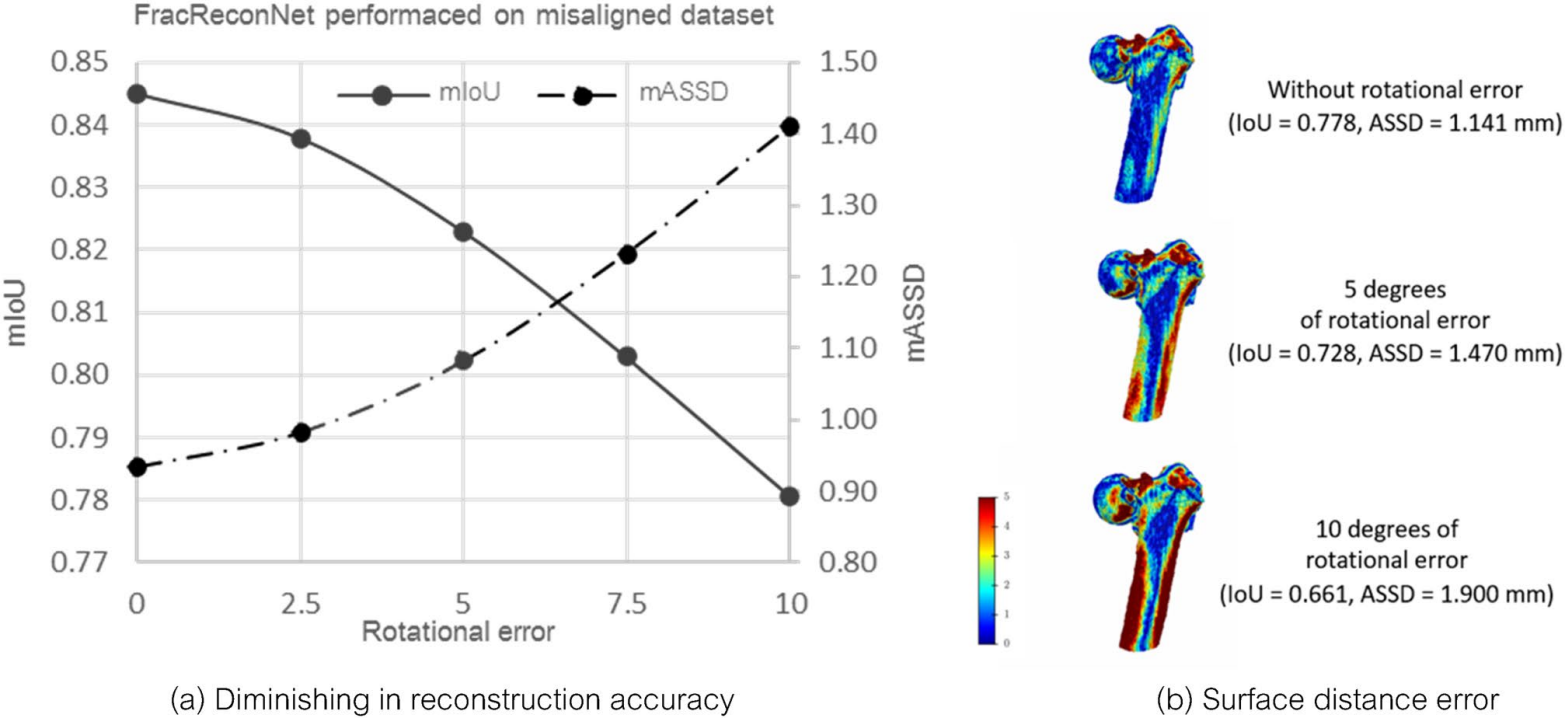
Effect of rotational error between two radiographs on reconstruction results.

Although our proposed method accomplishes fracture femur reconstruction, there are some usage limitations in the aspect of the radiograph imaging view. The method only supports the Judet view radiograph that takes −45° and + 45° on the vertical human axis. This imaging view avoids overlapping of the contralateral femur to the target fracture side^34^. However, it is a tough position to acquire the perfect orthogonal of each radiograph view. In the case of an X-ray machine supporting the lateral head tilt, the machine needs to be calibrated so that the ray of the X-ray beam is perpendicularly incident to the image detector^39^. If an X-ray machine does not support lateral head tilt, the patient needs to lie down on a 45° slope plain to acquire the Judet view imaging. A patient subjected to a femoral fracture may not be suitable for this position because of hurt at the fracture site. Moreover, the second view radiograph needs to be precisely 90° from the first view because the fusion module is capable only of orthogonal permutation. The non-orthogonal second view radiograph could affect the reconstruction result and unpromising fracture detail. From our experiment on the misalignment of biplanar radiographs, the mIoU and mASSD were gradually worse as the rotational error between two radiographs had risen (Fig. 7a). The mIoU and mASSD values had degraded (2.60%, 15.85%) and (7.57%, 50.96%) at 5° and 10° of rotational error, respectively. Figure 7b obviously showed sight of an increase in surface distance error likewise. This affected both overall outer surface and fracture region.

Finally, our proposed method dedicates only the 3D shape of the femur but no intensity. There is still a demand for the CT image-like with intensity in the Hounsfield unit. The Hounsfield unit is essential for bone quality assessment and prosthesis & fixation selection^40^. This problem requires massive training samples to gain more information for reconstruction. Acquiring massive CT images is costly and takes so much time for the data cleaning process. There is also no public CT fracture femur data to serve this problem.

## Conclusions

We have proposed the novel 3D reconstruction of proximal femoral fracture from biplanar radiographs called FracReconNet, including 3DReconNet, a novel auxiliary class, and fractural augmentation approach. Our experiments demonstrate that the FracReconNet is capable of reconstructing the 3D femur shape with fracture detail more precisely. Furthermore, the FracReconNet’s results show fracture details more similar to the real fracture, while the 3DReconNet cannot. The evaluation of FracReconNet achieved mIoU of 0.851 and mASSD of 0.906 mm. The FracReconNet has significantly improved mIoU and mASSD by 16.43% and 50.91%, respectively, compared to 3DReconNet. While it has improved over 3DReconNet-AC by 15.38% of mASSD. However, this proposed method was only validated in the laboratory, so it is in TRL-4 (technology readiness level). Therefore, using this method, users should be aware of rotational errors.

## Data Availability

The datasets generated and analyzed during the current study are not publicly available due to privacy and ethical restrictions but might be available on reasonable request from the corresponding author. The FracReconNet model and code implemented for this study are available here https://github.com/DanupongBu/FracReconNet.
